# The study of serum muscarinic acetylcholine receptor subtype 3 (m3AChR)-Specific autoantibodies level in rheumatoid arthritis patients with secondary sjogren syndrome

**DOI:** 10.1186/s41927-023-00368-1

**Published:** 2023-12-18

**Authors:** Hagar Elsayed Fakher, Nagat Mohammed El Gazzar, Maaly Mohamed Mabrouk, Doaa Waseem Nada

**Affiliations:** 1 Department of Rheumatology, Rehabilitation & Physical Medicine, Faculty of Medicine, Tanta University, El-Gharbia Governorate, Tanta City, Egypt; 2 Department of Clinical Pathology, Faculty of Medicine, Tanta University, Tanta City, El- Gharbia Governorate, Egypt

**Keywords:** Sjogren syndrome, Rheumatoid arthritis, Sensitivity, Specificity, Muscarinic acetylcholine receptor subtype 3 autoantibodies

## Abstract

**Background:**

Dry eyes and mouth are symptoms of Sjogren syndrome, which can occur on its own and be referred to as primary Sjogren syndrome or in conjunction with other rheumatic diseases like rheumatoid arthritis and be referred to as secondary Sjogren syndrome. Anti-muscarinic type 3 receptors have been linked to neurological issues as well as secretory dysfunction in Sjogren patients. Consequently, the purpose of this study is to determine the serum level of muscarinic acetylcholine receptor subtype 3 (m3AChR)-specific autoantibodies in rheumatoid arthritis (RA) patients and evaluate its relationship to disease activity, functional disability, and to study its role in the development of secondary Sjogren syndrome manifestations in those patients.

**Methods:**

In this cross-sectional study, 30 RA patients with secondary Sjogren syndrome signs and 30 RA patients without secondary Sjogren syndrome manifestations were included, along with 30 healthy volunteers who were aged, and sex matched as controls. All participants underwent thorough clinical examination, evaluation of disease activity using the DAS28 score, assessment of functional status using the modified health assessment questionnaire (MHAQ), and evaluation of the serum level of (m3AChR) by (ELISA).

**Results:**

When compared to RA patients without secondary Sjogren syndrome and healthy controls (20.09 ± 4.24, 18.36 ± 3.59 ng/ml respectively), the serum level of m3AChR antibodies among 30 RA patients with secondary Sjogren syndrome considerably increased (mean 25.98 ± 4.81 ng/ml).Analysis of the m3AChR’s (ROC)-curve revealed that the three groups under study differed significantly (*P* < 0.001), with the AUC (0.806), cutoff (> 22.63ng/ml), sensitivity (73.33%), and specificity (86.67%) all exceeding the threshold. Additionally, there was a significant positive connection between the serum level of m3AChR and the following variables (*P* < 0.05): DAS scores, MHAQ score, number of tender & swollen joints, and acute phase reactants.

**Conclusion:**

Autoantibodies against m3AChR may be one of the serum components involved in the pathophysiology of secondary Sjogren syndrome in RA patients, and because of their high sensitivity and specificity, they can be utilized as a diagnostic marker in these individuals.

## Introduction

Rheumatoid Arthritis (RA) is a chronic immune-mediated disease in which malfunctioning combination of immune cell types and signaling networks results in organ damage, primarily in the joints, and has a substantial negative influence on patients’ quality of life, mental state, and ability to function normally [1]. The worldwide prevalence of RA ranges from 0.3 to 1%; it is more frequent in women than in men (3:1 ratio); and its peak start occurs in the fourth and fifth decades of life [2].The primary symptom of RA is symmetric polyarthritis in the small joints of the hands, feet, and wrists, but it can also affect larger joints [3, 4].The chronic inflammatory state of RA can result in secondary Sjogren syndrome(sSS), which is present in roughly 19.5% of RA patients [5, 6].

Dry eyes and mouth are the most common symptoms of secondary Sjogren Syndrome (sSS), which can occur in conjunction with rheumatoid arthritis and increases its morbidity and mortality [7]. Other exocrine manifestations may include dryness of the nose, skin and vagina, recurrent sinusitis; recurrent oral candidiasis; chronic cough; frequent indigestion; and constipation [8]. The RA patients who are more susceptible to have sSS look like to be older, female, seropositive; has a longer disease duration; higher disease activity; and a higher incidence of comorbidities (hypertension, cardiovascular disease, malignancies, and serious infections), erosive disease, and extra-articular manifestations [6].

The histological characteristic of Sjögren’s syndrome (SS), lymphocytic infiltration of exocrine glands and epithelium, is assumed to be present in most patients [8]. Moreover, the exocrine glands have high levels of the muscarinic type 3 receptor (M3R), which when bound by muscarinic agonists like acetylcholine or carbachol, mediates the release of Ca2 + from intracellular calcium storage. It has been demonstrated that parotid acinar cells secrete saliva in response to this increase in intracellular Ca2 + concentration. Therefore, autoantibodies against the second loop of the M3R were found to reduce intracellular Ca2 + influx in functional investigation of human salivary gland cell lines, indicating suppression of saliva secretion and have been linked to neurological issues in SS patients [9, 10]. As, the presence of lymphocytic infiltration in salivary glands does not always indicate disease severity or the degree of secretory dysfunction [11]. Therefore, other potential factors like antibodies to muscarinic type 3 receptor (anti-M3R) contributing to SS dryness are actively investigated. Many researchers studied the clinical usefulness of Muscarinic type 3 receptor antibodies in primary Sjogren Syndrome(pSS) patients [6, 8, 12–14]. However, no other previous publications studied its diagnostic performance in secondary Sjogren Syndrome associated with RA patients. Subsequently, the purpose of this study is to determine the serum level of muscarinic acetylcholine receptor subtype 3 (m3AChR)-specific autoantibodies in RA patients and evaluate its relationship to disease activity, functional disability, and to study its role in the development of secondary Sjogren syndrome manifestations in those patients.

## Subjects and methods

### Study design & participants selection

This cross-sectional study included 60 rheumatoid arthritis patients fulfilling the classification criteria of the 2010 American College of Rheumatology ∕ European League Against Rheumatism [15]. All patients were selected from the outpatient clinics of Rheumatology, Rehabilitation and Physical Medicine Department, Faculty of Medicine, Tanta University, From May 2021 to June 2023 and were divided equally into two groups:

**Group I**: Included 30 RA patients with manifestations of secondary Sjogren syndrome & fulfilling the diagnostic criteria of 2002 American-European Consensus Group (AECG) [15]. Briefly, the existence of item 1 or item 2, plus any two from items 3, 4, and 5 is indicative of secondary SS: (1) Ocular symptoms, (2) Oral symptoms, (3) Ocular signs of Keratoconjunctivitis sicca (KCS), (4) Focal sialadenitis by minor salivary gland biopsy(focus score ≥ 1 per 4 mm^2^), (5) Objective evidence of salivary gland involvement, (6) Presence of autoantibodies (Anti Ro/SSA ,or Anti La /SSB or both ).

#### Group II

Included 30 RA patients without manifestations of secondary Sjogren syndrome.

In addition, thirty age and sex matched individuals from volunteers attending Rheumatology Outpatient clinic complaining of various musculoskeletal problems were also included as control group. None of the controls had a history of or symptoms compatible with other autoimmune diseases as xerophthalmia, xerostomia or Raynaud’s phenomenon.

### Exclusion criteria

The following cases were excluded from our study: (a) Patients with malignant diseases such as gastric carcinoma, hepatocellular carcinoma, breast cancer and prostate cancer. (b) Patient suffering from obesity as it was found that obesity induced by high-calorie diet strongly influences the expression and intracellular signaling coupled to M1-M3 mAChR subtypes. (c) Patient suffering from chronic infection as TB (d) Patient with Epilepsy.

### Ethics related considerations

This study was conducted in accordance with the Declaration of Helsinki and approved by the local ethical committee, Faculty of Medicine, Tanta University, Egypt **(approval code: (34,699/5/21))**. All patients understood the aim of the research and gave their informed consent for participation in this study. We followed the recommendations of STROBE guidelines during the preparation of this manuscript.

## Methods

All RA patients were subjected to the followings:

### Clinical assessment

Including full medical history taking, general examination and locomotor system examination,

#### Pain assessment

A 10 cm-length Visual Analog Scale (VAS) is used; one end of the scale denotes intensity 0 (no pain), and the other end denotes an intensity of 10 (unbearable pain) [16].

#### Testing for dry eye

Using paper strips inserted into the lower conjunctival sac, the Schirmer’s test was performed; a score of less than 5 mm in 5 min shows a tear deficiency [17].

#### Testing for dry mouth

Employing the Saxon test, which involves two minutes of chewing on a folded piece of sterile sponge. The measurement of saliva production involves the pre- and post-chewing weight of the sponge. The weight of the chewed sponge varied by more than or equal to 2.75 g in two minutes in the normal control subjects [18].

#### Assessment of disease activity

Utilizing Disease Activity Score in 28 joints (DAS 28) that measures disease activity in RA patient at a single point in time. It requires detecting the number of tender and swollen joints, patient global assessment of disease activity, and ESR [19].

### Functional assessment

Functional status was assessed using modified health assessment questionnaire (MHAQ). It retains one question in eight categories including: dressing and grooming, arising, eating, walking, hygiene, reaching, gripping, and other activities. The m HAQ score is then calculated as the mean of the scores for each activity [20].

### Laboratory assessment

#### Routine laboratory tests including

- Complete blood count (CBC) by automatic blood cell counter (ERMA) [21].
- Erythrocyte sedimentation rate (ESR) by Westergren tube method [22].
- C-reactive protein (CRP) by immunoprecipitation method [22].
- Rheumatoid factor (RF) by turbidimetric method [23].
- Anti–cyclic citrullinated peptide antibodies (Anti – CCP) by ELISA technique [24].
- Anti- Ro/SSA& Anti- La /SSB antibodies by ELISA technique [25].

### Specific laboratory tests including

Detection of Serum level of muscarinic acetylcholine receptor subtype 3 (m3AChR)- specific autoantibodies by ELISA technique using Human (CHRM3) kits (Sun Red, catalogue number 201-12-3471).

#### Blood sample collection

Blood collection was performed under sterile conditions, seven millimeters of venous blood were collected from each subject by use of disposable sterile plastic syringe, each sample was fractionated as follow: 2 ml of blood were collected in ethylene diamine tetra-acetic acid (EDTA)tube and mixed thoroughly to perform complete blood picture.2 ml were collected in sodium citrate 3.8% tube for ESR (1.6 ml blood with 0.4 ml citrate) & 3 ml were collected in plain tube, then centrifuged for 15 min at the 3000 rpm for separation of serum, then the serum was divided into two aliquots: One for determination of C-reactive protein, RF, anti-CCP, and anti- Ro/SSA& anti- La /SSB antibodies .The other aliquot was used for determination of (m3AChR)-antibodies.

#### Test principle

A double-antibody sandwich enzyme-linked immunosorbent assay (ELISA) was employed by the kit to measure the concentration of Muscarinic3 (CHRM3), the human cholinergic receptor, in samples. An enzyme that is pre-coated with a human monoclonal antibody against the cholinergic receptor, Muscarinic 3 (CHRM3), was added to the monoclonal antibody. After incubation, biotin-labeled antibodies against Cholinergic Receptor Muscarinic 3 (CHRM3) were added and mixed with streptavidin-HRP to create an immunological complex. After adding Chromogen Solution A, B, the liquid’s color first turned blue before eventually turning yellow due to the acid. There was a positive correlation between the sample’s content of the Human Substance Cholinergic Receptor Muscarinic3 (CHRM3) and its color chroma.

#### Assay procedure

**Fig. 1:**
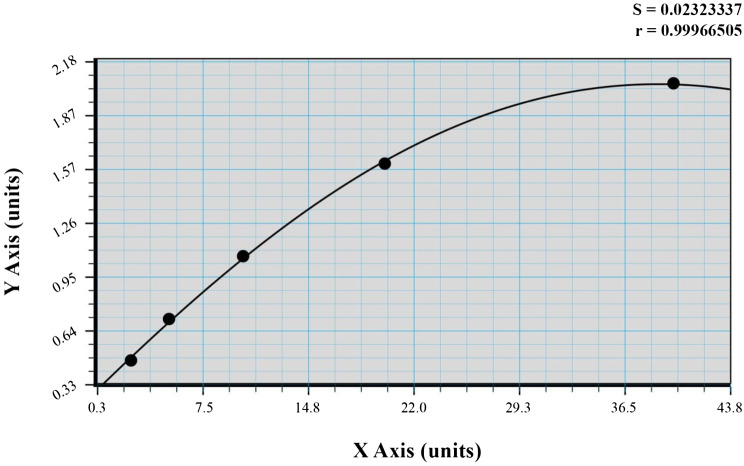
Sample curve

There was one original standard reagent included in this test kit. The number of standards and samples that need to be tested determines how many plates are needed. Every standard was made in duplicate and left blank. Stop solution, 40 µl of sample, 10 µl of CHRM3-antibody, and 50 µl of streptavidin-HRP were added after the chromogen solutions A and B. After sealing the membrane, it was gently shaken and incubated for 60 min at 37 °C. Following that, the membrane was gently removed, the liquid was emptied, and any leftover water was shaken off. Each well received 50 µl of chromogen solution A, 50 µl of chromogen solution B, and 10 min of incubation at 37 °C away from light. Next, 50 µl of Stop Solution was given to each well to halt the reaction. After applying the stop solution, the optical density (OD) was measured at 450 nm wavelength, with a blank taken as zero. This was done within 15 min. The linear regression equation was used to create the standard curve based on the concentration of the standards and the related OD values. The corresponding sample concentration was then determined by applying the sample OD values to the regression equation. We created the standard curve on graph paper using the standard density as the horizontal and the OD value as the vertical. The Sample curve, Fig. 1, was used to determine the corresponding density based on the sample OD value.

### Statistical analysis

Data was fed to the computer and analyzed using IBM SPSS software package version 20.0. Qualitative data were described using number and percent. The Shapiro-Wilk test was used to verify the normality of distribution. Quantitative data were described using mean& standard deviation, median and interquartile range (IQR). The Chi-square test was used to assess the qualitative data. When more than 20% of the cells have an anticipated count below 5, the Monte Carlo test was employed to correlate the chi-square statistic. The Mann Whitney U test was employed to compare qualitative variables between the two patient groups. The Pearson coefficient is used to determine the correlation between quantitative variables with normal distribution in the two studied groups. One-way ANOVA was also used to compare the two patient groups under study with the controls. Significance of the obtained results was judged at the 5% level. The receiver operating characteristic curve (ROC) was generated by plotting sensitivity (TP) on Y axis versus 1-specificity (FP) on X axis at different cut off values. The area under the ROC curve denoted the diagnostic performance of the test.

## Results

Table 1 displays the demographic information of the participants. The average age of RA patients in group I was (47.20 ± 9.91) years, whereas the average age of RA patients in group II was (44.53 ± 9.05) years. Most RA patients in both groups and the control group were female, accounting for (83.3%, 86.7%, and 76.7%) of the total. There was no significant difference in age or sex between the three groups (*P* = 0.076 & 0.073, respectively). Within the first group, 15 patients exhibited antibodies against Ro/SSA, 5 against La/SSB, and 10 against both La/SSB and Ro/SSA. On the other hand, neither the controls nor the patients in group II possessed antibodies against Ro/SSA or La/SSB.

**Table 1:** The demographic data of the participants

Variables	Group I (*n* = 30)		Group II (*n* = 30)		Control (*n* = 30)		Test of Sig.	*p*-value
	No.	%	No.	%	No.	%		
**Sex**								
Male	5	16.7	4	13.3	7	23.3	χ^2^= 5.284	^MC^*p* = 0.073
Female	25	83.3	26	86.7	23	76.7		
**Age (years)**								
Range	30.0–68.0		28.0–70.0		21.0–70.0		F = 2.652	0.076
Mean ± SD.	47.20 ± 9.91		44.53 ± 9.05		40.70 ± 13.50			

*SD*: *Standard deviation, F*: *One way ANOVA testχ*

^*2*^: *Chi square test*, *MC*: *Monte Carlo test*

*p* value for comparing between the three studied groups, *: Statistically significant at *p* ≤ 0.05

In terms of how long the disease has lasted, group I patients’ median duration of rheumatoid arthritis was insignificantly (*P* = 0212) longer than that of group II patients (8 years), whereas group II patients’ median duration of sicca symptoms was 6 months, with a range of 1 to 36 months. The mean DAS28 was (4.19 ± 1.53) for patients in group I and (3.91 ± 1.53) for patients in group II. Table 2 displays all the clinical information pertaining to the number of tender and swollen joints, the VAS, the duration of morning stiffness, and the MHAQ score in both groups of RA patients.

**Table 2:** Clinical characteristics of RA patients

Variables	Group I (*n* = 30)	Group II (*n* = 30)	Test of Sig.	*p*-value
**Duration of illness (years)**				
Range	2.0–45.0	0.50–30.0	U = 366.0	0.212
Median (IQR)	10.50 (5.0–18.0)	8.0 (4.0–15.0)		
**Duration of Sjogren manifestations (month)**				
Range	1.0–36.0	–	–	–
Median (IQR)	6.0 (3.0–12.0)	–		
**Xerophthalmia (** *n* **, %)**	30(100)	0(0)	--	--
Xerostomia (*n*, %)	30(100)	0(0)	--	--
Keratoconjunctivitis sicca (*n*, %)	24(80)	0(0)	--	--
Positive salivary gland biopsy (*n*, %)	10(33.3)	0(0)	--	--
Positive Saxon test (*n*, %)	27(90)	0(0)		
**Morning stiffness (min.)**				
Range	30.0–120.0	30.0–120.0	U = 386.50	0.305
Median (IQR)	55.0 (30.0–80.0)	50.0 (30.0–70.0)		
**Tender joints**				
Range	0.0–28.0	0.0–28.0	U = 416.0	0.614
Median (IQR)	8.0 (1.0–15.0)	7.0 (1.0–13.0)		
**Swollen joints**				
Range	0.0–5.0	0.0–6.0	U = 383.50	0.240
Median (IQR)	4.0 (0.0–8.0)	3.0 (0.0–6.0)		
**VAS**				
Range	10.0–80.0	10.0–100.0	U = 433.0	0.798
Median (IQR)	40.0 (30.0–60.0)	30.0 (20.0–60.0)		
**DAS28**				
Range	1.66–7.03	1.50–7.36	T= 0.705	0.483
Mean ± SD.	4.19 ± 1.53	3.91 ± 1.53		
**m HAQ**				
Range	0.0–2.28	0.0–2.28	U = 427.0	0.731
Median (IQR)	1.5 (0.1–2.28)	1.4 (0.1–2.28)		

*IQR*: *Inter quartile range, SD: Standard deviation*, *F*: *One way ANOVA testt*: *t- test*, *U*: *Mann Whitney test,χ*^*2*^: *Chi square test, MC: Monte Carlo test, VAS*: *visual analogue scale*, *DAS28*: *disease activity score in 28 joints*, *m HAQ* : *modified health assessment questionnaire*

As can be shown in Table 3, our analysis of the laboratory data revealed no statistically significant difference in ESR (*P* = 0.513) and CRP (*P* = 0.404) between RA patients in group I and II. Also, the levels of RF and anti-CCP in group I (median 80 IU/ml, 118 u/ml, respectively) and group II (median 50 IU/ml, 70.50 u/ml, respectively) were observed to differ significantly from one another. Additionally, the assessment of serum level of anti-M3R antibodies in our participants revealed significant (*P* < 0.001) increase of its level in RA patients in group I; (median 27.38 ng/ml) in comparison to RA patients in group II and healthy control (18.42, 17.26 ng/ml respectively), Table 3. Notably, anti- m3AChR was detected in group I patients with anti-SSA/Ro-negative (2/5, 40%), anti-SSB/La-negative (10/15, 66.7%).

**Table 3:** Laboratory characteristics of RA patients

Inflammatory markers	Group I (*n* = 30)	Group II (*n* = 30)	Control (*n* = 30)	Post Hoc test	*p*-value	Sig. bet. Groups.
**1st ESR (mm/h)**						
Range	7.0–75.0	9.0–65.0	5.0–10.0	44.937^*^	< 0.001^*^	p_1_ = 0.513 p_2_ < 0.001^*^ p_3_ < 0.001^*^
Median (IQR)	33.0 (20.0–40.0)	25.0 (18.0–37.0)	9.0 (8.0–10.0)			
**2nd ESR (mm/h)**						
Range	18.0–100.0	20.0–110.0	10.0–20.0	46.762^*^	< 0.001^*^	p_1_ = 0.553 p_2_ < 0.001^*^ p_3_ < 0.001^*^
Median (IQR)	60.0 (40.0–80.0)	50.0 (35.0–70.0)	18.0 (15.0–20.0)			
**CRP (mg/l)**						
Range	1.10–98.0	3.0–314.0	2.0–8.0	40.385^*^	< 0.001^*^	p_1_ = 0.404, p_2_ < 0.001^*^ p_3_ < 0.001^*^
Median (IQR)	24.45 (11.3–50.4)	18.0 (10.0–42.6)	5.0 (4.0–6.0)			
**RF (IU/mL)**						
Min. – Max.	6.0–512.0	1.0–240.0	1.0–20.0	55.101^*^	< 0.001^*^	p_1_ = 0.020^*^ p_2_ < 0.001^*^ p_3_ < 0.001^*^
Median (IQR)	80.0 (64.0–208.0)	50.50 (8.0–64.0)	3.0 (2.0–6.0)			
**Anti CCP (u/mL)**						
Min. – Max.	1.0–500.0	1.0–672.0	1.0–7.0	34.818^*^	< 0.001^*^	p_1_ = 0.005^*^ P_2_ < 0.001^*^ p_3_ < 0.001^*^
Median (IQR)	118.0 (70.0–324.0)	70.50 (24.0–207.0)	4.0 (2.0–5.0)			
**Positive Anti-Ro/ SSA (** *n* **, %)**	15(50)	0(0)	0(0)	--	--	--
**Positive Anti-La/SSB (** *n* **, %)**	5(16.7)	0(0)	0(0)	--	--	--
**Positive both Anti-Ro/ SSA& Anti-La/SSB (** *n* **, %)**	10(33.3)	0(0)	0(0)	--	--	--
**m3AchR (ng/ml)**						
Min. – Max.	16.08–32.89	14.32–33.79	14.18–25.98	26.640^*^	< 0.001^*^	p_1_ < 0.001^*^ p_2_ < 0.001^*^ p_3_ = 0.258
Median (IQR)	27.38 (21.51–29.47)	18.42 (17.49–21.65)	17.26 (14.82–20.63)			

*IQR: Inter quartile range, SD: Standard deviation, F: One way ANOVA test, pairwise comparison between. each 2 groups were done using Post Hoc Test (Tukey). p: p value for comparing between the three studied groups, p*_*1*_: *p value for comparing between Group I and Group II, p*_*2*_: *p value for comparing between Group I and control Group, p*_*3*_: p value for comparing between Group II and control Group. *: Statistically significant at p ≤ 0.05, ESR: erythrocyte sedimentation rate, CRP: C- reactive protein, RF: rheumatoid factor, Anti-CCP: Anti cyclic citrullinated peptide, m3AChR: muscarinic acetylcholine receptor subtype 3 specific autoantibodies

**Fig. 2:**
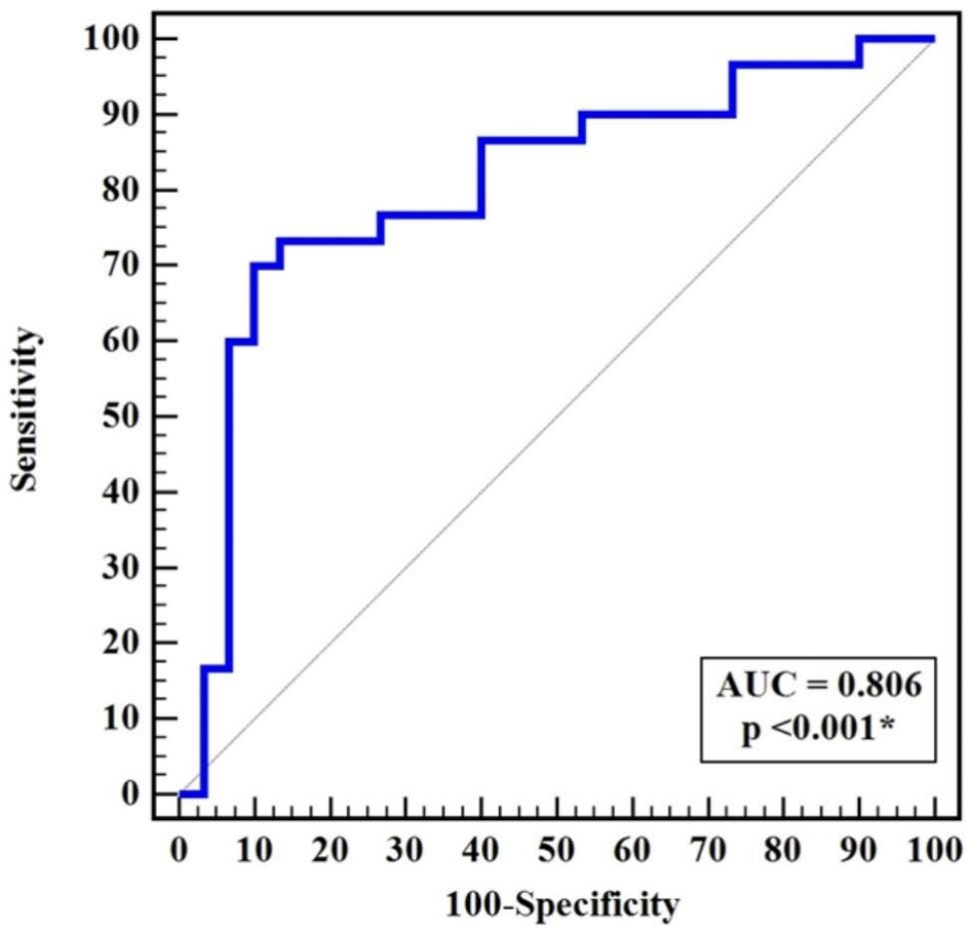
ROC curve of m3AChR antibodies

The m3AChR test’s cutoff value was (> 22.63ng/ml). The AUC (0.806), Highly significant difference between the studied groups (*P* < 0.001), sensitivity (73.33%) and specificity (86.67%) in Table 4 and (ROC)-curve analyses, Fig. 2, demonstrated its good diagnostic value. Additionally, our research demonstrated a positive and statistically significant link between the serum level of m3AChR and the following variables: anti-SSA/Ro (*P* = 0.0234), VAS (*P* = 0.026), ESR (*P* = 0.014), CRP (*P* = 0.014), number of tender joints (*P* = 0.001), swollen joints (*P* = 0.018), DAS scores (*P* = 0.003), and MHAQ scores (*P* = 0.003), Table 5.

**Table 4:** Diagnostic performance of m3AChR autoantibodies

	AUC	*p*	95% CI	Cut off^#^	Sensitivity	Specificity	PPV	NPV
**m3AChR**	0.806	< 0.001^*^	0.689–0.922	> 22.63	73.33	86.67	84.6	76.5

*AUC: Area Under a Curve, p value: Probability value, 95% CI: 95% Confidence Intervals*

NPV: Negative predictive value, PPV: Positive predictive value *: Statistically significant atp ≤ 0.05

*#Cut off was choose according to Youden index*

**Table 5:** Correlation between m3AChR autoantibodies with different clinical and laboratory parameters in group I

m3AChR	*r*	*p*
**DAS-28 score**	0.523	0.003^*^
**MHAQ**	0.102	0.003^*^
**Morning stiffness (min.)**	0.128	0.501
**Tender joints**	0.559	0.001^*^
**Swollen joints**	0.429	0.018^*^
**Duration of Sjogren (/month)**	0.265	0.157
**Duration of illness (years)**	0.135	0.477
**1st ESR**	0.444	0.014^*^
**2nd ESR**	0.453	0.012^*^
**CRP**	0.257	0.014^*^
**RF**	0.265	0.158
**Anti CCP**	0.006	0.973
**Anti Ro/SSA**	0.211	0.023*
**VAS**	0.407	0.026^*^

r: Pearson coefficient *: Statistically significant at p ≤ 0.05, ESR: erythrocyte sedimentation rate, CRP: C- reactive protein, RF : rheumatoid factor, Anti-CCP: Anti cyclic citrullinated peptide, m3AChR: muscarinic acetylcholine receptor subtype 3 specific autoantibodies ,VAS: *visual analogue scale*, *DAS28: disease activity score in 28 joints*, *m HAQ* : *modified health assessment questionnaire*

## Discussion

Secondary Sjogren’s syndrome is one of most frequent extra-articular manifestations, that occurring in approximately 30% of RA patients, where involvement of exocrine lacrimal and salivary glands occurs [26]. The presence of autoantibodies against muscarinic acetylcholine receptor subtype 3(m3AChR) has been reported in primary Sjogren syndrome patients, and it is suggested that an immune reaction to (m3AChR) plays a crucial part in the pathogenesis of SS [27]. Although many studies proved the role of m3AChR antibodies in the pathogenesis of primary Sjogren syndrome, there are not enough studies about its role in secondary Sjogren syndrome. Hence, the aim of our study was to assess the serum level of (m3AChR)-specific autoantibodies in RA patients, investigate whether its level in RA patients is correlated with the presence of secondary Sjögren Syndrome manifestations, and determine its relation to disease activity, severity, and functional disability.

A total of 90 participants were divided into three groups: 30 RA patients with secondary Sjogren syndrome manifestations, 30 RA patients without secondary Sjogren syndrome manifestations, and 30 healthy volunteers who were age- and sex-matched to the patients were included in our study. Nearly most of RA patients with or without sSS, and the control group were females (83.3%, 86.7% and 76.7% respectively) with insignificant difference between the three studied groups as regard age & sex (P = 0.076 & 0.073 respectively). This is in line with **Haga** et al. [28]., who examined the prevalence of Sicca symptoms and secondary Sjögren’s syndrome in 300 rheumatoid arthritis patients, there were no differences in RA patients with and without sSS in terms of age and sex. While **Harrold** et al. [29]., who conducted an observational study from the Corrona registry for the prevalence of Sjögren’s syndrome associated with rheumatoid arthritis in the USA reported more prevalence of sSS among older and more likely female patients. They included 7870 RA patients with sSS in the study, and they claimed that the large number of participants helped to detect the difference in age and sex between the patients with sSS and those without. However, the result that middle-aged women make up most of patients in the two groups may be explained by the fact that RA is more prevalent in middle-aged women, which is related to female sex hormones and more responsive immune system. The feminine predominance may also be supported by genes in sexual chromosomes [30]. Similar to rheumatoid arthritis, it is hypothesized that Sjogren’s syndrome is associated with a decline in estrogen levels. Estrogen, which is crucial for female immunity, is thought to act as a protective mechanism against rheumatoid arthritis and Sjogren’s syndrome because higher levels can reduce inflammation by increasing regulatory cytokines like interleukin-10 (IL-10) and transforming growth factor- (TGFB) [31].

Also, there was no significant difference between the two studied patient groups in terms of disease duration and morning stiffness, and the majority of our RA patients had moderate disease activity, and functional disability. These findings are in accordance with those of **Antero** et al. [32]., and **Gucey** et al. [33], who examined secondary Sjogren’s syndrome in rheumatoid arthritis patients and discovered no statistically significant difference between patients with RA and those without sSS in terms of disease activity or quality of life.

This is also in line with the findings of **Haga** et al. [28], and **El-Barbary** et al. [26], who discovered no variations in the illness duration between RA patients with and without sSS. **Zhang** et al. [34], who compared the therapeutic response between RA patients with SS and those without found that sSS is associated with lower probability of reaching remission or low disease activity in RA patients and should be regarded as one of the poor prognostic factors. However, some studies, such as **He** et al. [35], proved that RA patients with sSS had higher disease activity score. Additionally, **Jensen** et al. [36], showed that RA patients with sSS had higher MHAQ scores when examining the features of rheumatoid arthritis patients with self-reported sicca symptoms.

Regarding the impact of disease duration on the prevalence of sicca symptoms, **Harrold** et al. [29], reported a higher prevalence of sSS among RA patients with longer disease duration. They attributed this finding to the large sample size of their study, which made it possible to identify the difference in disease duration between patients with or without sSS. Many studies proved that the presence of sSS, one of the more common extra articular manifestations, does not correlate with disease activity, even though extra articular manifestations suggest an aggressive course of rheumatoid arthritis and do not diminish over a 10-year follow-up even when disease activity is controlled [37].

As regard acute phase reactants, there was no significant difference between RA patients with SS and those without, this was in line with **El-Barbary** et al. [26]., who found non-significant difference between RA patients with sSS and those without sSS, according to DAS 28 and laboratory parameters, including ESR, CRP. The fact that all patients (both those with RA and those with RA with sSS) were receiving first-line treatment exclusively with nonsteroidal anti-inflammatory, corticosteroid, and disease-modifying agents may have had an impact on the lack of difference, according to **Oleivira** et al. **‘s** [38] findings that CRP and ESR did not differ between those with RA and those with RA with sSS. Also, we found Most of RA patients had positive RF and Anti-CCP with significant increased titers in RA patients with sSS in comparison to RA patients without sSS.

This conclusion is consistent with research by **Brown** et al. [39], who examined the clinical features of RA patients with secondary SS and its relationship to joint degeneration. They found that individuals with sSS had higher RF and ACPA titers. Researchers, **Turesson** et al. [40], and **Rycke** et al. [41], examined the relationship between rheumatoid factor and antibodies to cyclic citrullinated peptides and severe extra-articular manifestations in rheumatoid arthritis. They discovered that rheumatoid factor is strongly associated with these manifestations, while the relationship between anti-CCPs and these manifestations is similar but weaker. While **El-Lopez** et al. [42], who investigated the relationship between anti-CCP and anti-mutated citrullinated vimentin (anti-MCV) antibodies and extra-articular manifestations in rheumatoid arthritis and **Haga** et al. [28], who reported no statistically significant difference in RF and anti-CCP antibody levels in RA patients with sSS and those without sSS.

In group I, 15 of our patients had anti-Ro/SSA antibodies, 5 had anti-La/SSB antibodies, and 10 patients had both anti-Ro/SSA and anti-La/SSB antibodies. In contrast, neither the patients in group II nor the controls had anti-Ro/SSA or anti-La/SSB antibodies. Moreover, we found a significant difference in m3AChR antibodies level between RA patients with sSS and those without, with remarkable increase in patients with sSS, and there is also significant difference between them and control group, while there was no significant difference between RA patients without sSS and control group. Only anti-SSA/Ro was significantly associated with antiM3R frequency (*P* = 0.0234). Moreover, anti-M3R was detected in group I patients with anti-SSA/Ro-negative (2/5, 40%), anti-SSB/La-negative (10/15, 66.7%),

This is consistent with the findings of **Tsuboi** et al. [43], who discovered that SS patients had much greater frequencies and titers of anti-M3R antibodies than the control group did. These antibodies target all extracellular domains. Additionally, **Bacman** et al. [44], observed mAChR was considerably higher in the plasma of pSS and sSS patients in comparison to non-SS patients with sicca symptoms and RA patients without sicca symptoms, revealing a similar conclusion. **Zuo** et al. [45], in their study on 18 RA, 23 heathy control, 24 pSS, and 18 SLE patients concluded that both anti-SSA/Ro and anti-M3R reflect a critical distribution and are most likely to enhance noninvasive identification of individuals with SS .Furthermore, a portion of SS patients with negative anti-SSA/Ro, anti-SSB/La, were positive for anti-M3R suggesting some individuals may benefit from inclusion of anti-M3R testing for SS diagnosis.

On contrary, **Hatipoğlu** et al. [46], found that antibodies against the first, second, and third extracellular loop (M3R^211–230^) were not increased in anti-SSA positive patients compared to healthy controls in connective tissue diseases.

It is well known that autoantibodies to the m3AChRs function as an agonist-like agent that stimulates the parasympathetic nervous system and activates the synthases enzymes (NOS). It is also possible that in pSS and sSS, direct m3AChR antibody-mediated tissue damage may occur through nitric oxide generation and accumulation, which has a negative impact on the lacrimal and salivary glands by its cytotoxic effects on the cell [47].

The m3AChR can considerably predict secondary Sjogren in RA patients with high specificity (86.67%) and intermediate specificity (73.33%), according to the analysis of the Roc curve in our study.

When researching m3AChR antibodies in pSS, **Zuo** et al. [45], discovered that their sensitivity was 75% and specificity was 95.6%. As a result, although the sensitivity of m3AChR antibodies in pSS is comparable to that in sSS in this investigation, it appears to be more specific in pSS. Additionally, we discovered a strong positive association between the level of m3AChR and the following variables: acute phase reactants, DAS scores, MHAQ, number of sore and swollen joints, and VAS. These findings are consistent with those of **Marlia** et al. [48], who investigated the impact of xerostomia on the functional ability of rheumatoid arthritis patients and discovered a positive and substantial link between the number of painful joints, HAQ, and DAS28, as well as sSS symptoms.

Except for our study, there have been no other explanations of the function of m3AChR antibodies in sSS as a diagnostic marker or demonstrations of the relationship between m3AChR antibodies and clinical and serological characteristics in sSS patients.

**However, this study had some limitations**, the first was the small sample size of each group, which might have impacted the accuracy of the findings. The second was that we used AECG criteria to diagnose sSS, which depend on subjective symptoms of ocular and oral dryness, and we excluded pSS patients as a group to study the differences in m3AChR antibodies between pSS and sSS.

## Conclusion

The m3AChR antibodies may play a potential role in the pathogenesis of secondary Sjogren Syndrome associated with RA and can be used as a diagnostic marker as it has high sensitivity and specificity. Moreover, RA patients with negative anti-SSA/Ro, or anti-SSB/La may benefit from inclusion of anti-M3R testing for detection of secondary Sjogren Syndrome. To investigate the variations in m3AChR antibody sensitivity and specificity between pSS and sSS, additional multicenter studies with large sample sizes are required.

## Data Availability

The datasets generated and/or analyzed during the current study are not publicly available due to [privacy / ethical restrictions] but are available from the corresponding author on reasonable request.
